# Advances for Pulmonary Functional Imaging: Dual-Energy Computed Tomography for Pulmonary Functional Imaging

**DOI:** 10.3390/diagnostics13132295

**Published:** 2023-07-06

**Authors:** Yoshiyuki Ozawa, Yoshiharu Ohno, Hiroyuki Nagata, Keigo Tamokami, Keitaro Nishikimi, Yuka Oshima, Nayu Hamabuchi, Takahiro Matsuyama, Takahiro Ueda, Hiroshi Toyama

**Affiliations:** 1 Department of Radiology, Fujita Health University School of Medicine, Toyoake 470-1192, Aichi, Japan; 2 Department of Diagnostic Radiology, Fujita Health University School of Medicine, Toyoake 470-1192, Aichi, Japan; 3 Joint Research Laboratory of Advanced Medical Imaging, Fujita Health University School of Medicine, Toyoake 470-1192, Aichi, Japan

**Keywords:** dual energy, CT, spectral, lung, functional, pulmonary embolism

## Abstract

Dual-energy computed tomography (DECT) can improve the differentiation of material by using two different X-ray energy spectra, and may provide new imaging techniques to diagnostic radiology to overcome the limitations of conventional CT in characterizing tissue. Some techniques have used dual-energy imaging, which mainly includes dual-sourced, rapid kVp switching, dual-layer detectors, and split-filter imaging. In iodine images, images of the lung’s perfused blood volume (PBV) based on DECT have been applied in patients with pulmonary embolism to obtain both images of the PE occluding the pulmonary artery and the consequent perfusion defects in the lung’s parenchyma. PBV images of the lung also have the potential to indicate the severity of PE, including chronic thromboembolic pulmonary hypertension. Virtual monochromatic imaging can improve the accuracy of diagnosing pulmonary vascular diseases by optimizing kiloelectronvolt settings for various purposes. Iodine images also could provide a new approach in the area of thoracic oncology, for example, for the characterization of pulmonary nodules and mediastinal lymph nodes. DECT-based lung ventilation imaging is also available with noble gases with high atomic numbers, such as xenon, which is similar to iodine. A ventilation map of the lung can be used to image various pulmonary diseases such as chronic obstructive pulmonary disease.

## 1. Introduction

Dual-energy (DE) imaging, which was introduced as a first-generation dual-source computed tomography (CT) system in 2006, can improve the differentiation of material by using two different X-ray energy spectra [1,2]. DE imaging has provided a new imaging approach for diagnostic radiology with its ability to overcome the limitations of conventional CT for characterizing tissue [3,4,5,6,7,8,9,10,11,12,13,14]. For example, dual-energy CT (DECT) reconstructions provide iodine maps for analyses of perfusion, virtual unenhanced images, virtual non-calcium images, or virtual monochromatic or monoenergetic images (VMI) [3,10,13,15,16,17]. With the increasing availability of DECT systems, DECT is becoming a multiparametric analyzer and enhances diagnostic accuracy in the thoracic area, especially in vascular diseases.

In this article, we review the basics of DECT, its clinical applications, and its value for pulmonary functional imaging.

## 2. Basics of Dual-Energy CT 

### 2.1. Basic Principles of Dual-Energy CT

In conventional single-energy CT, the X-ray’s attenuation of tissue (linear attenuation coefficient or CT number) depends on the effective energy of the polychromatic X-ray beam, the material’s density, and the material’s effective atomic number (Zeff) [4,12,18]. Thus, a material has different CT numbers at different energy levels [2,3,5], and the degree of the difference depends on the elemental composition of the material. In conventional single-energy CT, materials with different atomic numbers can have similar CT numbers due to their mass density, which makes it difficult to differentiate materials (e.g., calcium and iodine). On the other hand, acquisition at different energies in DECT allows the differentiation of material due to the different attenuation characteristics of tissues at different X-ray energies [12,15,18]. The DE ratio of the iodinated contrast material at tube voltages of 80 and 140 kVp is approximately 2 (600 HU/300 HU) and that for calcified bone is 1.5 (800 HU/533 HU). Thus, the DE ratio can differentiate iodine from other high-attenuation structures such as calcium and bone [15]. In theory, the two different energy data points are acquired to solve two equations and obtain two unknown values (a_p_ and a_c_), which are the coefficients describing the contributions of the photoelectric and Compton effects, respectively, and only depend on the atomic number of the tissue [4,19]:

µ(LE) = a_p_f_p_(LE) + a_c_f_c_(LE) (1)

µ(HE) = a_p_f_p_(HE) + a_c_f_c_(HE) (2)

where µ(LE) and µ(HE) are the attenuation coefficients for low- and high-energy X-ray beams, and f_p_(LE), f_c_(LE), f_p_(HE), and f_c_(HE) are known mathematical functions that depend only on the energy of the photon beam. 

### 2.2. Dual-Energy CT Scanners

There are some clinical DECT scanners: dual-source CT, rapid kVp switching CT, dual-layer detector CT, and split-filter CT. Two independent X-ray energy spectra, low (70–100 kVp) and high (135–150 kVp), are obtained using these scanners for DECT imaging [4]. Two independent X-rays are used for dual-source CT and rapid kVp switching CT, while dual-layer detector CT and split-filter CT use only one X-ray source, and the beam is separated into low- and high-energy spectra at the level of the detector or at the tube’s output [3,4,5,11,12,20]. Table 1 shows a summary of various techniques of dual-energy imaging [3,4,5,10,12,20,21]. There is also another approach of DE imaging, namely dual-spin or sequential scans with a single tube, which needs the least hardware and acquires DE data by switching the tube’s voltage in two sequential scans in axial or helical mode [10,11,12,20]. The limitation of dual-spin or sequential scans is the temporal delay between the two acquisitions, which causes the image to degrade, especially for rapidly moving structures such as the heart [12].

There are two methods of DE analysis: raw-data-based (projection-data-based) and image-based methods [12,22,23]. The raw-data-based method processes the projection data obtained directly from the CT acquisition. On the other hand, the image-based method processes the images after reconstructing the high- and low-energy images. Raw-data-based spectral reconstruction requires spatial and temporal alignment of the low- and high-energy data. Therefore, the raw-data-based method can be used with rapid kVp switching CT and dual-layer detector CT [5,12]. Processing DECT using the image-based method is applicable in dual-source CT and split-filter CT, which do not have spatiotemporal registration. The low- and high-energy sinograms are separately reconstructed into low- and high-energy images in this image-based method. This is followed by decomposition of the material to generate two density maps of the base material. Image-based decomposition of the material has a greater number of beam-hardening artifacts, which can be reduced by iterative beam-hardening correction in the image domain [12]. In CT scanning, the phenomenon of beam hardening is caused by the preferential attenuation of low-energy X-ray photons rather than high-energy X-ray photons as polychromatic X-rays pass through an object. This can cause streaks and dark bands, especially after passing through highly attenuated areas such as dense bone, metallic devices, and areas with a high concentration of the contrast medium [5,17,24]. The raw-data-based DE analysis achieves fewer beam-hardening effects and fewer artifacts related to the reconstruction kernel of CT [9]. This analysis results in more accurate CT measurements of the number of scanned objects. In raw-data-based analysis, beam hardening is corrected during the generation of the material’s projection data from the original projection data. DECT images are less affected by beam-hardening artifacts, and their analysis is more accurate than image-based analysis [25,26]. 

#### 2.2.1. Dual-Source CT

Dual-source CT has two independent source-detector systems, which are mounted on the same gantry at an offset of about 90° or 95° [1,11,17]. The projection data collected using the dual-source systems are in a double-helix geometry, in which the two helical trajectories have a phase difference of approximately 90° and the projections from the low- and high-energy scans are not coincident with each other [22,23]. An image-based algorithm is required for DE image reconstruction. 

The noise levels in the images are adjustable because dual-source CT can choose the tube’s voltage and current independently for both tubes [3,10,20]. An additional filer such as a tin filter can be applied to the high-energy tube to filter out low-energy photons and enhance the spectral separation between the two energy spectra [10,11,12]. Therefore, dual-source CT has relatively better spectral separation.

Dual-source CT has limitations, as follows. First, significant photon cross-scattering occurs when two X-ray tubes operate simultaneously, and this increases the spectral overlap and noise, although implementing scatter-correcting algorithms during image reconstruction have been developed to correct for the increased projection noise arising from photon cross-scattering [27]. Second, the significant discrepancy in the view angles between the low-energy and high-energy projection sets could affect information related to the decomposition of the material, especially when motions of the patient caused by rapidly moving structures such as the heart or lung occur during the two acquisitions [10,11]. Third, in a dual-source CT scanner which has two independent source-detector systems, the second detector is restricted to a scan with a small field of view (FOV) (26, 33, and 35.5 cm, respectively, for each generation). On the other hand, the first detector has a 50 cm scanning FOV. This system results in limiting the acquisition and reconstruction FOV to 26–35.5 cm [4]. This limitation becomes a problem when scanning relatively large patients.

#### 2.2.2. Rapid kVp Switching CT

This method is based on the implementation of a single X-ray tube which can rapidly alternate between low and high voltages for each X-ray projection [10,11,12,20]. The switching of the tube’s voltage occurs very rapidly (approximately every 0.2 ms) in this system. Therefore, the low- and high-energy projection sets are acquired from practically the same view angle. Temporal misregistration can be negligible due to the near-simultaneous acquisition of projection data at both voltages, and spectral reconstruction in both the projection and image domains is possible [11,26]. This raw data (projection data) domain can achieve more accurate correction of beam-hardening in the measured projections and decomposition of the material [11,28].

There are limitations related to rapid kVp switching CT, namely the gantry’s rotation speed and the overlap of the spectral data. The rotational speed of the CT system is reduced to account for the acquisition of these additional projections and the rise and fall times of the modulation of voltage. Therefore, the gantry’s rotation time usually must be 0.5 s or longer, which results in prolongation of the acquisition time and leads to motion artifacts [20]. Overlap of the spectral data occurs because the process of switching the voltage is not instantaneous and the same filter is utilized for both energy levels [12].

#### 2.2.3. Dual-Layer Detector CT

This system has a single-X ray tube and a layered detector set [11,12,20]. The layered detector is composed of a top layer of an yttrium-based garnet scintillator, which absorbs most of the low-energy photons, and a bottom layer of gadolinium oxysulfide, which absorbs most of the high-energy photons [11]. Dual-layer detector CT has an advantage that the low- and high-energy projection data can be acquired simultaneously [11,12], and the spatial and temporal resolution of DE imaging is excellent. Spectral reconstruction in both the raw data (projection data) and image data domains is possible in this system. This system does not need to select DECT protocols in advance, as the acquisition of data is always in DECT mode. Therefore, DE imaging can be performed retrospectively after CT scanning in routine practice [3,10]. Dose modulation techniques, a full FOV (50 cm), and a full rotation speed can also be utilized. 

Dual-layer detector CT has the limitation of a relatively high overlap between the energy spectra. The sensitivity profiles of the scintillator materials between the two layers overlap considerably. This system has lower sensitivity to optical photons and crosstalk between the two detector layers [10,26]. The mechanism of this DE data-acquisition method relies on the assumptions that all the low-energy photons are attenuated in the top detector layer and that the high-energy photons do not interact with the material in the top layer during the transit to the bottom layer of the detector [11]. Violation of these assumptions causes suboptimal separation of the spectra between the two energy levels and inaccurate decomposition of the material [11]. 

#### 2.2.4. Split-Filter CT

In this system, a filter is used to split an X-ray beam into two half-beams with different spectra [10,11,13]. A split filter is placed in front of the X-ray tube, which is composed of gold and tin stacked adjacent to each other in the longitudinal direction to achieve spectral separation of a polychromatic X-ray beam [11]. When a 120 kVp X-ray beam passes through the filter, two beams with different mean photon energies of 67.5 keV (gold) and 85.3 keV (tin) are emitted from the halves of the filter. The gold has a role as a beam softener and tin as a beam hardener [11,12]. These low- and high- energy spectra are detected by the corresponding halves of the detector system along the longitudinal (z) direction [12]. As an advantage of this system, many clinical CT scanners can be upgraded by adding a split filter for DE imaging. A full FOV (50 cm) is also available. However, split-filter CT has suboptimal temporal registration of low- and high-energy data because each voxel is imaged using two different energy spectra at different times [12]. The ability for spectral separation is relatively low [29]. Greater X-ray output is necessary because prefiltration absorbs approximately two-thirds of the radiation, although the radiation dose is similar to that of single-energy CT [10]. 

## 3. Clinical Application of Dual-Energy CT for Pulmonary Functional Imaging

### 3.1. Iodine Imaging

#### 3.1.1. Perfused Blood Volume in the Lung

DECT can visualize the iodine components in tissues [7,8,30,31]. Through use of a three-material decomposition algorithm, iodine images or iodine-enhanced images can be generated [5]. A perfused blood volume (PBV) image or pulmonary blood volume image represents the distribution of iodine in the lung’s parenchyma [5,8,30,31,32,33,34]. PBV images show the contrast enhancement of the lung’s parenchyma at a single time point but can be used as a surrogate for perfusion [8]. 

**Figure 1:**
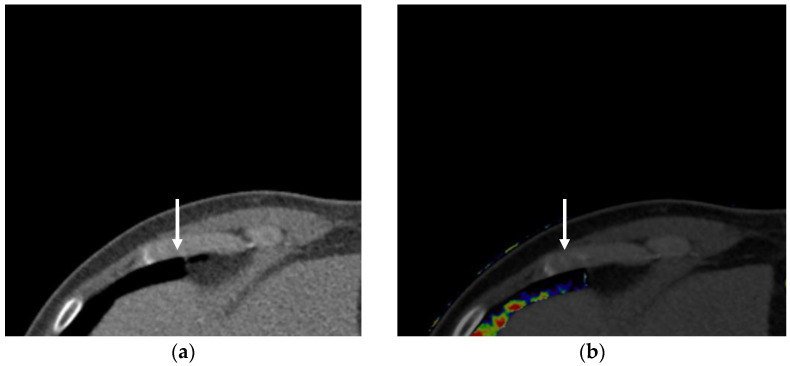
An acute pulmonary embolism: (**a**) CT pulmonary angiography; (**b**) Image of perfused blood volume in the lung. The clot (arrow) in the pulmonary artery is difficult to identify because of the lack of spatial resolution in CT pulmonary angiography. The image of the perfused blood volume images shows the perfusion defect in the affected lung area (arrows), which can improve diagnostic accuracy for detecting a pulmonary embolism.

Diagnosing a pulmonary embolism (PE) is one of the main clinical applications of PBV imaging. In routine practice, conventional CT pulmonary angiography (CTPA) is predominantly used to detect a PE, because of its easy accessibility, short acquisition time, direct depiction of intra-arterial clots, and additional information other than PE [35]. Owing to the subsequent development of multidetector CT with improvements in the spatial and temporal resolution and serial improvements in the quality of the images, CTPA has basically achieved high accuracy in diagnosing PE. However, for various reasons, including a lack of spatial resolution [36], respiratory motion, image noise in larger patients, or beam-hardening artifacts caused by medical devices, a proportion of studies remain suboptimal [8], and CTPA has limited ability for detecting small emboli in the segmental and subsegmental arteries [36,37]. Images of the lung’s PBV based on DECT have been applied in patients with PE to simultaneously obtain images of both the PE occluding the pulmonary artery (PA) and the consequent perfusion defects in the lung’s parenchyma [7]. The PBV of the lung can show more information than CTPA only, which compensates for the lack of spatial resolution of CTPA [38] (Figure 1).

Chronic thromboembolic pulmonary hypertension (CTEPH) is a disease caused by the persistent obstruction of pulmonary arteries by organized thrombi, which leads to redistribution of the flow and secondary remodeling of the microvascular bed [35]. Among the various diseases which cause pulmonary hypertension (PH) [35], CTEPH is curable if the organized thrombus is completely resected by surgery (pulmonary endarterectomy). Balloon pulmonary angioplasty and pharmacological treatments are also included in the therapy for CTEPH [35]. Lung ventilation–perfusion (V/Q) scintigraphy is one of the important tools for diagnosing pulmonary hypertension including CTEPH [39]. The interpretation of the findings of a V/Q scan is not difficult in patients with CTEPH, and the reported sensitivities and specificities are 96–97% and 90–95%, respectively [35]. Most patients with CTEPH have obvious abnormal findings from the V/Q scan and often have multiple moderate to large perfusion defects without matching ventilation defects in their lungs [39,40,41].

**Figure 2:**
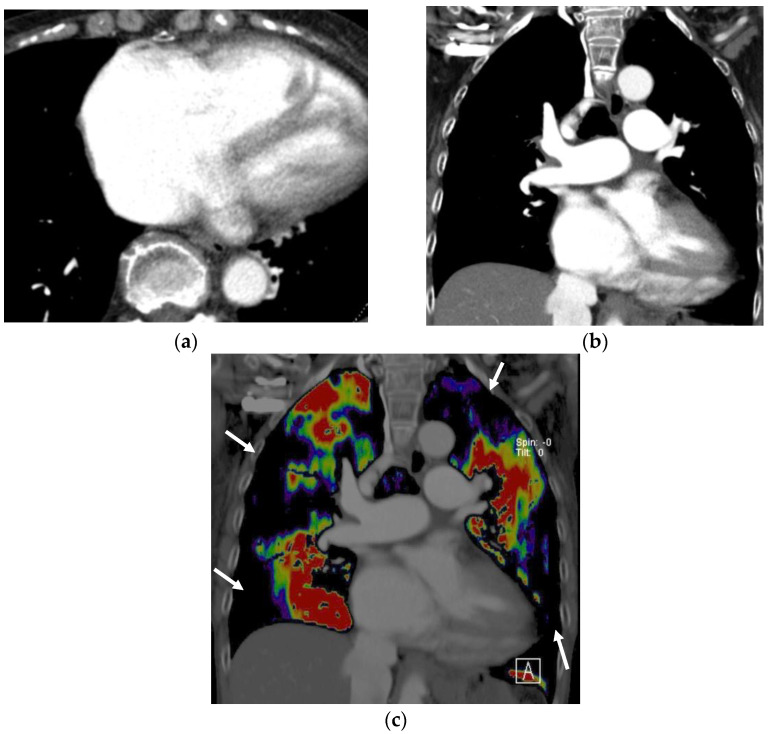
Images of the perfused blood volume in the lung of a patient with chronic thromboembolic pulmonary hypertension: (**a**) Transverse CT pulmonary angiography at the level of the heart demonstrates an enlargement in the right ventricle, which indicates right heart failure. (**b**) Coronal CT pulmonary angiography demonstrates dilatation of the main pulmonary artery. Conventional CT pulmonary angiography does not show information on lung perfusion. (**c**) The image of the perfused blood volume in the coronal lung, corresponding to image (**b**), demonstrates multiple perfusion defects (arrows) in the bilateral lung, which are typical findings of chronic thromboembolic pulmonary hypertension.

For the detection of CTEPH at the level of segmental pulmonary arteries, the overall diagnostic performance for V/Q scanning and CTPA was similar (area under the curve (AUC): 0.67 for V/Q planar scintigraphy, 0.64 for V/Q single photon emission computed tomography (SPECT), and 0.64 for CTPA) [42]. V/Q SPECT was significantly more sensitive than CTPA for detecting pulmonary artery obstructions (85% vs. 67%), while the specificity was significantly greater for CTPA than for V/Q scanning (60% vs. 42%, respectively) [42]. In a comparison of DECT with V/Q planar scintigraphy or V/Q SPECT, good agreement between both images was demonstrated [32,33]. For the diagnosis of CTEPH, the sensitivity, specificity, positive predictive value (PPV), and negative predictive value (NPV) have been reported to be 0.97, 0.86, 0.85, and 0.97, respectively, for images of PBV of the lung based on DECT, while values of 0.97, 1, 1, and 0.98 were found for V/Q scans, and there was excellent agreement between the images of PBV of the lung and scintigraphy for the diagnosis of CTEPH (kappa value = 0.80) [33] (Figure 2). In other studies, strong quantitative correlations for relative lobar perfusion, which was calculated by dividing the amount of the radiotracer or iodinated contrast agent per lobe by the total amount in both lungs, were found between images of the lung’s PBV and V/Q SPECT-CT, and the R^2^ of the linear regression analysis of mean relative lobar perfusion for both modalities was 0.87 [43]. With respect to SPECT-CT as the reference standard, the sensitivity, specificity, PPV, NPV, and accuracy for assessments of qualitative lobar perfusion defects based on PBV images were 89.4%, 96.5%, 95.6%, 91.4%, and 93.0%, respectively, and a high level of agreement was found for the morphology and severity of perfusion defects between both modalities (kappa = 0.84 and 0.86, respectively) [43]. Moreover, diagnosis using both PBV images and CTPA could correct the false positive and negative cases of DECT perfusion [33]. Recent publications evaluating PBV images in CTEPH demonstrated levels of sensitivity and specificity above 0.95, while some earlier studies had lower specificity due to streak artefacts [8]. Streak artifacts derived from high concentrations of the contrast media in the superior vena cava and subclavian vein can cause defects in the PBV images around these structures, which can result in false positive diagnoses of PE [30]. The systemic collateral supply in chronic PE can be one of the factors that can affect the detection of PE or perfusion defects in the lung by PBV images [30,44,45,46,47]. Systemic collateral blood flow to the lung has been shown to increase by as much as 30% relative to the original blood flow after PA occlusion [47]. This systemic collateral circulation increases significantly in chronic PE compared with acute PE and can be the cause of the distinct pulmonary perfusion patterns observed in patients diagnosed with chronic PE [48]. DECT demonstrates PBV images which reflect not only the supply of the PA but also the systemic artery’s supply because the injected iodine contrast medium also can pass into the lung via collateral systemic circulation, especially when the scan time is long or the scanning delay is not appropriately set for the early phase. This is a point of difference between lung perfusion scintigraphy using a radioactive tracer of ^99m^Tc-macroaggregated albumin (^99m^Tc-MAA), which reflects the first pass of the PA’s flow into the lung because the MAA is trapped in the pulmonary capillary bed. Dual-phase contrast-enhanced DECT can be used to evaluate differences in the regional perfusion patterns between patients with acute PE and those with chronic PE. It has been reported that the lung segments affected by chronic PE were significantly more enhanced in the delayed phase of dual-phase DECT than those of acute PTE. Therefore, dual-phase evaluation of the lung’s parenchyma suggests that acute and chronic PE can be differentiated by the infilling of the initial PBV defects in chronic PE compared with the absence of infilling in acute PE [48], which may be caused by the development of systemic collateral formations in chronic PE [48]. In one study, enlarged collateral systemic arteries in the patients with chronic pulmonary thromboembolism were observed more significantly and frequently ipsilateral to the lungs with perfusion defects (75%) compared with lungs without perfusion defects (23%). DECT demonstrated an association between the severity of perfusion defects and the degree of development of the systemic collateral supply [49]. However, in a study that evaluated right heart catheterization and dual-phase DECT [50], correlations between the morphological findings of the collateral bronchial arteries and the variables obtained via the dual-phase DECT in patients with PH were also investigated. Although enlarged bronchial arteries were more common in the patients with PH, the presence of moderate to markedly prominent collateral arteries did not correlate with the increase in volumetric whole-lung enhancement in delayed phase DECT, which had the best correlation with pulmonary vascular resistance (PVR) among the DECT-based parameters [50]. Thus, the main reason for delayed lung enhancement in patients with PH may be the altered pulmonary arterial perfusion due to structural hemodynamic changes in the pulmonary arteries themselves, although the presence of systemic blood flow from the bronchial arteries may be a contributory factor [8].

Quantification of PBV in the lung based on DECT can be assessed, and there has been some research into the association of the severity of PE or PH and the number of perfusion defects [16,34,51,52,53,54,55,56,57]. In conventional CT, some CT findings such as dilatation of the PA and the right ventricle (RV), have been observed in patients with PH [58,59,60,61,62]. In patients with CTEPH, it has been reported that the main pulmonary arterial to ascending aortic diameter ratio measured using contrast-enhanced CT had an association with the risk of first clinical exacerbation, and the ratio of the diameter of the right ventricle (RV) to that of the left ventricle (LV) was associated with the risk of poor prognosis in inoperable CTEPH [59]. In Kaplan–Meier analysis, there were significant differences in hospitalization in the patients with different main PA to ascending aortic diameter ratios with a cutoff level of ≥1.1, and in the patients with different RV/LV diameter ratios with a cutoff level of ≥1.2. There was a significant difference in the prognosis between the patients with an RV/LV ratio of ≥1.2 and those with an RV/LV ratio of <1.2 [59]. In other studies using CT pulmonary angiography, the prognostic value and cutoff levels of the imaging parameters were as follows: for PE-related mortality, the odds ratio (OR) and 95% confidence interval (CI) were 5.0 and 2.7–9.2 at an RV/LV diameter ratio of ≥1.0, and for all-cause mortality, the hazard ratio (HR) and 95% CI were 6.5 and 1.8–23.8 at an RV/LV volume ratio of >1.2, respectively [35,60,61]. Contrast reflux in the inferior vena cava is also a robust method for predicting 30-day mortality in patients with acute PE [62]. On the other hand, the degree of perfusion defects obtained using images of PBV in the lung based on DECT can be a new imaging parameter for estimating the severity of pulmonary arterial diseases, including CTEPH [52,55,57], PH [54], and acute PE [34,51,53,56]. Regarding CTEPH, it has been reported that the extent of PBV defects detected using DECT significantly showed positive correlations with the mean PA pressure and PVR [55,57], although some divergent results showed that the PBV in the lung was positively correlated with mean PAP [52]. 

Regarding acute PE, the relative volume of perfusion defects (volume of perfusion defects/total lung volume × 100) was also associated with an increased risk of death from any cause within 30 days (HR: 1.04) and pulmonary embolism-related death (HR: 1.05) [53]. It has been also reported that patients with adverse clinical outcomes showed a significantly higher volume of perfusion defects (35 ± 11% vs. 23 ± 10%, *p* = 0.002) [56]. The quantified PBV in the lung in the patients at a high and intermediate risk was significantly lower than that in the control group, and the PBV in the lungs of patients with acute PE had a negative correlation with the RV/LV diameter ratio (*R* = –0.567, *p* < 0.001) [51].

#### 3.1.2. Virtual Monochromatic Imaging

**Figure 3:**
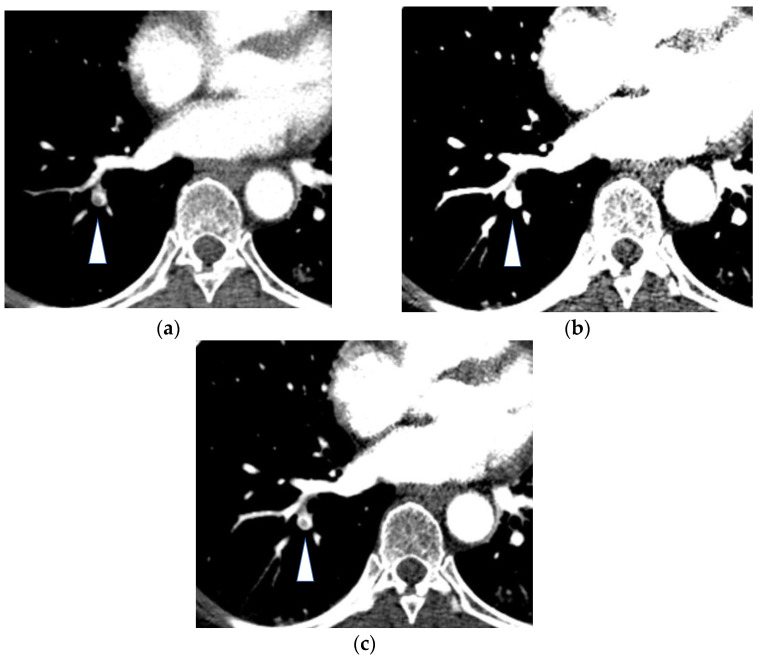
Acute pulmonary embolism (arrowheads) in the segmental artery of the right lower lobe: (**a**) Transverse CT pulmonary angiography at 120 kVP; (**b**) Noise-optimized virtual monochromatic image at 40 keV in which the window settings were the same as in (**a**); (**c**) Noise-optimized virtual monochromatic image at 40 keV in which the window settings were adjusted to depict the embolus in the pulmonary artery. The CT numbers in the pulmonary artery are 289 HU in (**a**) and 840 HU in (**b**,**c**). The virtual monochromatic image increased the contrast enhancement of iodine.

Another advance of DECT is that it can generate virtual monochromatic imaging (VMI) or monoenergetic imaging [5,13,15,22,36]. The X-ray beams are composed of photons in a wide continuum of energy (kiloelectronvolt, keV). Therefore, the beams are referred to as polychromatic X-rays that form the X-ray spectrum. The maximum value of the energy of photons in the X-ray spectrum matches the X-ray tube’s kilovolt peak (kVp). For example, a tube voltage of 100 kVp means the maximum energy of the spectrum is 100 keV [5,20]. The term “virtual” in VMI is used because the X-ray tubes for medical imaging do not produce a single level of energy but instead irradiate the patient with a wide range of X-ray energies. VMI is an image that simulates the CT images obtained using monochromatic X-rays with an arbitrary level of energy [5] ranging from 40 to 200 keV, which depends on the DECT scanners. The CT attenuation number at approximately 65–70 kiloelectronvolts (keV) in VMI is equivalent to the number in single-energy CT scans acquired at 120 kVp [22]. Thus, VMI in the energy range of 65–70 keV is selected as the standard image. In general, the image noise of VMI in this energy range is the lowest [22]. VMI at lower keV, e.g., 40–60 keV, can increase the attenuation of the iodine contrast medium [12,16,22,63,64,65]. In particular, as the energy level shifts close to the k-edge of iodine (36 keV), VMI at 40 keV can substantially increase the vascular contrast (Figure 3) [66].

**Figure 4:**
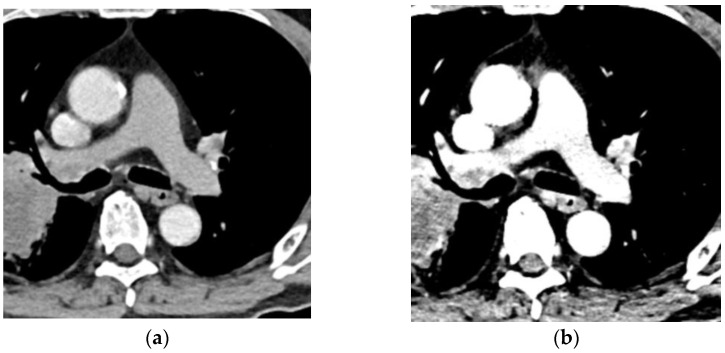
CT pulmonary angiography with a 50% reduction in the contrast medium: (**a**) Transverse CT pulmonary angiography at 120 kVp; (**b**) Noise-optimized virtual monochromatic image at 40 keV. The CT numbers in the pulmonary trunk are 95 HU at 120 kVP and 268 HU at 40 keV. The virtual monochromatic image can salvage the insufficient contrast enhancement caused by the reduced volume of contrast medium.

Increased enhancement of iodine contrast by using low keV VMI enables an evaluation of the peripheral subsegmental PA with more confidence [36,67]. In association with this benefit, it is important to set an optimized window width/levels for low-keV VMI DECT angiography [15,68,69] because low-keV VMI considerably increases the vascular attenuation, and the standard window setting is not appropriate for evaluations of the vascular structures. It has been reported that the optimized window width/levels for noise-optimized 40-keV VMI were 1070/380 in DECT pulmonary angiography and 1350/430 in DECT abdominal angiography [68,69]. Low-keV virtual monochromatic images may salvage the insufficient contrast enhancement of the PA [10], which is caused by various factors, such as inappropriate scan timing, a reduction in the amount of the contrast medium due to renal dysfunction, and dilution of the contrast medium by an influx of unenhanced inferior vena cava blood flow into the right heart, especially in the situation of the Valsalva maneuver associated with deep inspiration [6,13,65,70,71]. For this reason, low-keV VMI at 40–50 keV derived from DECT can allow for a reduction in the dose of the contrast material of around 50% (Figure 4) [72,73]. This technique is especially beneficial for patients with renal dysfunction. Low-keV VMI can be also used to improve the vessels’ contrast and to detect incidental pulmonary embolism in patients with portal-venous phase CT scans by substantially increasing the contrast to noise ratio and the signal to noise ratio [74].

As the trade-off of increases in the contrast enhancement of iodine, image noise tends to be increased in VMI at lower keV [22,66,75]. As a method of the noise reduction, the frequency-split method is used to extract the desired frequency information from two sets of images acquired at low and optimal energy levels for VMI reconstructions, and they are subsequently combined to produce a new series of images of the same frequency showing the characteristics of high-contrast and low-noise [76].

#### 3.1.3. Iodine Imaging for Thoracic Oncology

Iodine imaging also has the potential to improve clinical management in the area of thoracic oncology, e.g., differentiating between benign and malignant pulmonary nodules, characterizing mediastinal lymph nodes, and evaluating the response to therapy [30,36,77,78].

It is sometimes difficult to differentiate malignant pulmonary nodules from benign nodules in conventional CT. It has been reported that the CT attenuation numbers for DECT-based iodine-enhanced images with a cutoff value of 20 HU for malignant nodules had higher sensitivity and accuracy than the numbers using the degree of enhancement in blended images (sensitivity, 92% and 72%; specificity, 70% and 70%; and accuracy, 82.2% and 71.1%, respectively) [77,79]. In other research, although the degree of contrast enhancement in malignant nodules was significantly lower than that in inflammatory nodules, all parameters of DECT-based iodine uptake (total iodine uptake volume, total iodine concentration, and the iodine uptake volume and concentration without the necrotic part) in malignant nodules were significantly higher than those in inflammatory nodules (*p* < 0.001) [80]. VMI also can allow the characterization of tissues by evaluating their attenuation with X-rays at consecutive energy levels, which can assess the slope of the spectral curves of tissues and could be helpful for differentiating tissues that cannot be distinguished using conventional CT [78,81]. The slope of the spectral curves based on VMI had a strong ability to differentiate malignant nodules from benign ones, with the area under the curve being 0.86 in the arterial phase and 0.89 in the venous phase [81].

Adenocarcinoma in situ (AIS) generally presents as a persistent pure ground glass nodule (GGN) in CT. However, minimally invasive adenocarcinoma (MIA) and invasive adenocarcinoma also can present not only as part-solid nodules but also pure GGNs [82,83]. The 5-year disease-free survival rate for invasive adenocarcinomas is inferior to that of preinvasive lesions, and it is important to accurately differentiate them [83]. Iodine imaging can evaluate the degree of contrast enhancement in a GGN [83,84]. When the modified iodine concentration (IC), which was calculated as (IC of pure GGN—IC of ipsilateral normal lung tissue)/IC of ipsilateral normal lung tissue, was measured in pure GGNs, the modified IC in invasive adenocarcinomas and MIAs was significantly higher than that in preinvasive lesions such as AIS (0.45 ± 0.11 and 0.20 ± 0.14, respectively (*p* = 0.000)), although the simple IC itself did not show a significant difference between them (*p* = 0.054) [83].

Iodine imaging also provides additional information for characterizing the mediastinal and hilar lymph nodes in the N staging of lung cancer [77,85,86]. It has been reported that the IC or the slope of the spectral curve could differentiate between metastatic lymph nodes and nonmetastatic ones [85,86]. Low-keV VMI can improve the delineation between hilar lymph nodes and the adjacent pulmonary vessels, which is helpful for evaluations for N staging in lung cancer [87]. The contrast between the PA and lymph nodes was highest at the early phase (scan delay: 20 s) of the 120 kVp images, but the contrast of 40-keV VMI at the delayed phase (scan delay: 60 s) was second highest in the evaluated images. The contrast between the lymph nodes and the pulmonary veins was similar between these two images. Furthermore, beam-hardening artifacts derived from the contrast medium in the superior vena cava that are generally seen in the early phase of contrast-enhanced CT can affect the depiction of hilar lymph nodes. However, 40-keV VMI in the delayed phase could reduce the beam-hardening artifact derived from the contrast medium in the superior vena cava [87].

Assessments of the treatment response by CT are generally based on serial measurements of the tumor’s size [77]. Iodine imaging has the potential to evaluate the response of lung cancer to treatment [88].

### 3.2. Xenon Imaging

DECT-based imaging of lung ventilation uses noble gases with high atomic numbers, such as xenon [89]. The atomic number of xenon is 54, which is similar to that of iodine (atomic number 53), and the X-ray absorption characteristics of xenon resemble those of iodine [30]. Ventilation maps of the lung can be acquired by inhaled xenon-enhanced DECT because xenon is distributed in a similar way to air, and is detectable and quantifiable using DECT with three-material decomposition [7,10,30,67,90]. Xenon-enhanced DECT is clinically used to evaluate some situations or diseases, such as predicting residual pulmonary function after segmentectomy/lobectomy, detecting bronchiolar disease in asthmatic patients or lung transplant recipients, and mapping ventilation in patients with chronic obstructive pulmonary disease [91,92,93,94].

## 4. Conclusions

DECT provides additional functional information compared with single-energy CT for evaluations of the thoracic area. There are several dual-energy CT scanners, which have various characteristics with advantages and disadvantages. In the thoracic area, new techniques of DE imaging, which mainly include images of PBV in the lung, VMI, and ventilation maps have substantial potential to facilitate the assessment of the functional information of the lung.

## Figures and Tables

**Table 1:** Summary of various techniques of dual-energy imaging.

Techniques of DE Imaging	Dual-Source	Rapid kVp Switching	Dual-Layer Detector	Split-Filter
Number of X-ray sources	2	1	1	1
Number of detectors	2	1	1, layered	1
FOV (cm)	26, 33, 35.5 *	50	50	50
DECT analysis methods	Image-based	Raw-data-based	Raw-data-based	Image-based
Spectral separation	Good	Good	Good	Limited
Cross-scatter	Yes	No	No	Yes
Tube filtration	Yes	No	No	Yes
Tube current optimization for each energy bin	Yes	No	No	No
Temporal registration for DE imaging	Fair	Good	Excellent	Poor

Notes. DE, dual energy; kVp, kilovolt peak (tube voltage); FOV, field of view; DECT, dual-energy computed tomography. * FOV depends on the generation of the dual-source CT.

## Data Availability

Not applicable.
